# Aortic Valve Calcium Scoring Using True and Virtual Non-Contrast Reconstructions on Photon-Counting CT with Differing Slice Increments: Impact on Calcium Severity Classifications

**DOI:** 10.3390/tomography11120139

**Published:** 2025-12-11

**Authors:** Mandeep Singh, Amirhossein Moaddab, Doosup Shin, Jonathan Weber, Karen Chau, Ali H. Dakroub, Roosha Parikh, Karli Pipitone, Ziad A. Ali, Omar K. Khalique

**Affiliations:** 1 Department of Cardiology, St Francis Hospital & Heart Center, Roslyn, NY 11576, USA; 2 College of Osteopathic Medicine, New York Institute of Technology, Old Westbury, NY 11568, USA; 3 Division of Cardiovascular Imaging, St. Francis Hospital and Heart Center, Roslyn, NY 11576, USA

**Keywords:** aortic valve calcium score, photon-counting detector-computed tomography, virtual non contrast, true non contrast, slice thickness, computed tomography

## Abstract

**Background/Objectives**: Aortic valve calcification is commonly evaluated using 3.0 mm true non-contrast (TNC) computed tomography (CT) images. This study evaluates the reproducibility of virtual non-contrast (VNC) reconstructions at different slice intervals using photon-counting detector CT (PCD-CT). **Methods**: In this retrospective study, we included 279 consecutive patients, who underwent PCD-CT for evaluation of native aortic valve between February 2023 and December 2023 with both TNC and VNC images at 3.0 and 1.5 mm slice intervals. Aortic valve calcium score (AVCS) and aortic valve calcium volume (AVCV) were compared between the two methods using paired *t*-tests. Agreement for continuous variables was assessed using inter-class coefficients (ICCs). Cohen’s Kappa (κ) was calculated to evaluate the agreement between different modalities in diagnosing severe AV calcification. **Results**: Compared to the standard, TNC images at 1.5 mm intervals showed higher AVCS (mean difference: −290 ± 418, *p* < 0.001), with high reproducibility between techniques (CS: ICC 0.969, [IQR 0.962, 0.975]). Compared with reference, VNC showed no significant differences in AVCS at either slice intervals, with excellent reproducibility (3.0 mm, ICC 0.970 [0.963, 0.976]; 1.5 mm, ICC 0.971 [0.964, 0.977]). Compared to TNC 3.0 mm, strong concordance was observed using other reconstruction techniques in assessing severe AV calcification (κ = 0.81 [95% CI: 0.74–0.88], 0.83 [95% CI: 0.76–0.90], and 0.83 [95% CI: 0.76–0.90] for TNC at 1.5 mm, VNC at 3.0 mm, and 1.5 mm, respectively), with low misclassification rates. **Conclusions**: Our study highlights high reproducibility in the evaluation of AVCS by VNC reconstruction at 3.0 and 1.5 mm intervals compared with reference offering a reliable alternative with an excellent diagnostic accuracy.

## Simple Summary

In our study, we compared virtual non-contrast (VNC) images with true non-contrast (TNC) scans for measuring aortic valve calcium (AVC) at both 3.0 mm and 1.5 mm slice thicknesses using photon-counting CT. We showed strong reproducibility of VNC scans relative to standard TNC across different slice thicknesses. Our results demonstrated VNC as a reliable alternative for AVC assessment. In the future, incorporating VNC into routine clinical practice may help reduce radiation exposure while still providing comparable evaluation of valvular disease.

## 1. Introduction

Aortic valve calcium (AVC) is a common cause of aortic stenosis, with increasing prevalence in an aging population. The progressive thickening of the valve leaflets can lead to decreased cardiac output and heart failure due to increased mechanical stress on the left ventricle [1]. The degree of AVC, measured in Agatston units (AU), is closely linked to disease severity and is proportional to cardiovascular complication risk [2]. While transthoracic echocardiography is preferred for the initial assessment of aortic stenosis, computed tomography has emerged as a complementary tool for an accurate AVC quantification [3,4].

Photon-counting detector computed tomography (PCD-CT) is a novel technology offering several advantages over traditional scintillation detectors. PCD-CT directly converts X-ray quanta into electrical signals proportional to absorbed energy, enabling spectral CT and offering advantages such as reduced electronic noise and finer spatial resolution than conventional CT [5]. Prior to transcatheter aortic valve repair (TAVR), an additional scan is required for the aortic valve calcium score (AVCS) quantification using the true non-contrast (TNC) CT. Images are typically acquired using 3.0 mm slice intervals and infrequent use of 1.5 mm increments, with kV values ranging from 120 to 140 [6]. Virtual non-contrast (VNC) images can be derived from contrast-enhanced CT datasets by removing iodine from the contrast images, potentially reducing radiation exposure. While there is good consensus on the reliability of VNC images for coronary calcium analysis, data on their use for AVC quantification remain scarce [7]. This study aims to evaluate the reproducibility and reliability of VNC images compared to standard TNC 3.0 mm for AVC evaluation at different slice intervals.

## 2. Materials and Methods

### 2.1. Study Population

**Figure 1:**
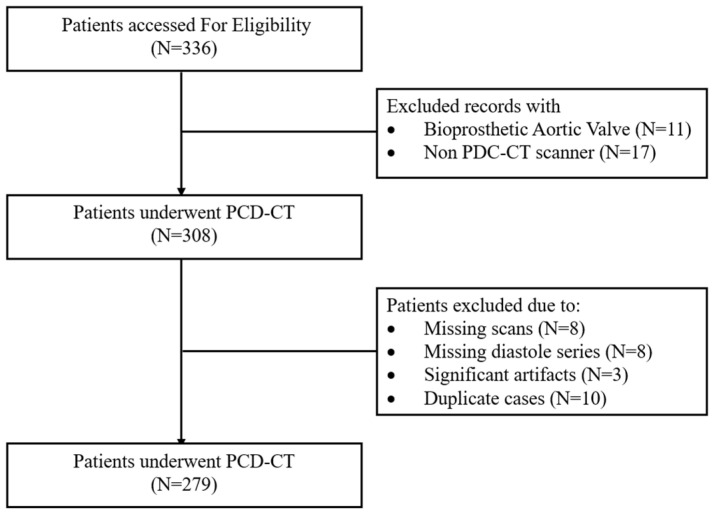
Patient flowchart demonstrating patient selection process. PCD-CT: photon-counting detector computed tomography.

This single-center, retrospective study included consecutive patients aged 18 years or older, who underwent cardiac computed tomography (CTA), using PCD-CT (Siemens NAEOTOM Alpha, with Syngo.CT version VA50A software, Siemens Healthineers, Forchheim, Germany,) as part of their pre-TAVR workup between February 2023 and December 2023. The indications for PCD-CT included comprehensive assessment of the aortic root and annulus for device sizing, evaluation of coronary ostial height, and sinus anatomy to assess the risk of coronary obstruction, and quantification of AVC. Fifty-seven patients were excluded due to missing TNC or VNC scans, prior valve-in-valve or bioprosthetic valve procedures, or missing diastolic series (Figure 1). Baseline demographics and clinical characteristics were collected via chart review. The study protocol adhered to the principles of the Declaration of Helsinki, followed the Strengthening the Reporting of Observational Studies in Epidemiology (STROBE) guidelines [8], and was approved by the Institutional Review Board of St. Francis Hospital and Heart Center, with a waiver of informed consent due to minimal risk.

### 2.2. CCTA Image Acquisition and Reconstruction Methods

TAVR CTA and coronary artery calcium score (CACS) scans were acquired on the Siemens Naeotom Alpha PCD-CT scanner. All CT acquisitions were ECG-gated, with end-diastole and end-systole defined according to the ECG-based cardiac cycle. Non-contrast CACS was performed using 120 kVp in FLASH mode and was automatically reconstructed at mid-diastole using a QR36 kernel with a quantum reconstruction level of 2. Then, 3 mm slices were reconstructed using both 3 mm and 1.5 mm slice increments. TAVR CTA was acquired using 140 kVp in sequential mode, and VNC images were reconstructed using a QR36 kernel, with a quantum reconstruction level of 2 and a 70-keV monoenergetic setting at both 3 mm and 1.5 mm intervals. For the post-contrast dataset, the diastolic phase matching closely to mid-diastolic timing of the non-contrast scan was selected for analysis.

### 2.3. Assessment of the AVCS

Quantification of the AVCS and aortic valve calcium volume (AVCV) was performed independently by two dedicated researchers. The assessment was made using 3mensio Structural Heart 10.6 SP2 (Pie Medical Imaging, Maastricht, The Netherlands) on both TNC and VNC reconstructions at 3.0 mm and 1.5 mm slice intervals, and AVCS and AVCV were calculated using manual segmentation of the aortic valve leaflets over sequential axial views [6]. Images were evaluated first in orthogonal planes (axial, coronal, and sagittal views) to ensure accurate assessment of contiguous valvular calcium confined to the AV leaflets and immediate annulus. Non-AV calcium was excluded, including any calcium in the aortic sinus, coronary arteries, mitral valve, aortic wall, and left ventricular outflow tract [9].

### 2.4. Statistical Analysis

Continuous values were reported as mean ± standard deviation or median (interquartile range [IQR]), and categorical values were presented as frequency (%). We compared AVCS and AVCV between and within reconstruction methods following recommended guidelines for conducting reproducibility/repeatability analyses around the technical performance assessment of imaging biomarkers [10]. Comparisons between the reconstruction methods were performed using TNC 3.0 × 3.0 mm as the reference standard. Bland–Altman plots were created to demonstrate agreement between/within reconstruction methods displaying mean bias and 95% limits of agreement (LOA). Log transformations were employed to account for the notable heteroscedasticity of AVCS and AVCV (i.e., log(AVCS + 1)), and Bland–Altman analyses were repeated with log-transformed data [11]. Scatter plots are used to display the direct relationship between reconstruction methods as well as predictive linear model parameters. Inter-observer comparisons (ICC) were performed using the concordance correlation coefficient, with 95% confidence intervals (CI) on both original and log-transformed data, and mean differences were compared using paired *t*-tests or Wilcoxon’s rank-sum test as appropriate. AVCS was dichotomized and considered high when >2000 AU for males and >1200 AU for females [12,13]. Cohen’s Kappa (κ) along with sensitivity/specificity was calculated to estimate agreement between categories of high/low AVCS. Analyses were performed using SAS version 9.4 (SAS, Inc., Cary, NC, USA), with plots created using R version 4.4.2.

### 2.5. Post Hoc Analyses

Two random samples of 15 subjects each were used to test the inter-observer reproducibility between the two researchers and the intra-observer reproducibility with themselves, respectively, 1 month after the initial image analysis was complete, to ensure variability within/between operators did not significantly impact the primary reconstruction method comparison. We additionally performed an examination of proportionality bias using Bland and Altman’s method [14] by regressing the average log-transformed AVCS against the measurement difference (y- and x-axis of the Bland–Altman plot) for each reconstruction comparison. The test for proportionality bias is equivalent to the global test for the slope/beta estimate under the null hypothesis of no proportionality bias (beta = 0). Gender-stratified comparisons of the reconstruction methods were produced to evaluate potential gender differences.

## 3. Results

### 3.1. Study Population

**Figure 2:**
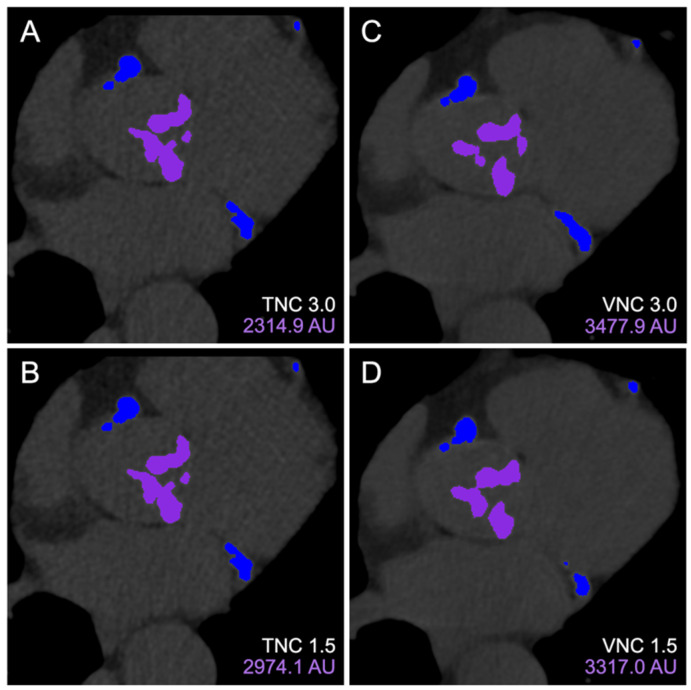
Aortic valve calcium scoring on 3mensio Structural Heart. Calcium contours of the same cross-section were visualized on TNC 3.0 mm (**A**), TNC 1.5 mm (**B**), VNC 3.0 mm (**C**), and VNC 1.5 mm (**D**) reconstructions with purple and blue overlay mask demonstrating locations of included aortic valve and excluded coronary artery calcifications, respectively. Abbreviations: TNC, true non-contrast; VNC, virtual non-contrast.

There were 279 patients included in this study from February to December 2023 (Figure 1). The median age was 80 years (IQR 74–86), and 46% were female. Every patient underwent cardiac CT, with PCD-CT as part of the TAVR workup. Figure 2 demonstrates an example subject, with significant AVC (2315 AU) measured using all four reconstruction methods.

The median BMI was 27.8 kg/m^2^ (IQR 24.5–31.5), and the majority of patients had a history of hypertension (94%) or hyperlipidemia (76%). Roughly one-third of the patients had a history of diabetes (33%) or atrial fibrillation (28%). The median total contrast volume was 70 mL (IQR 60–70), and the peak tube voltage for the contrast images was set at 140 kVP per the manufacturer’s recommendation (Table 1). The median total AVCS was 1957 AU (IQR 1182–2954). Among males, 66% had a total AVCS greater than 2000 AU, while 57% of females had a total AVCS exceeding 1200 AU.

### 3.2. Concordance and Discordance Between Reconstruction Methods

**Figure 3:**
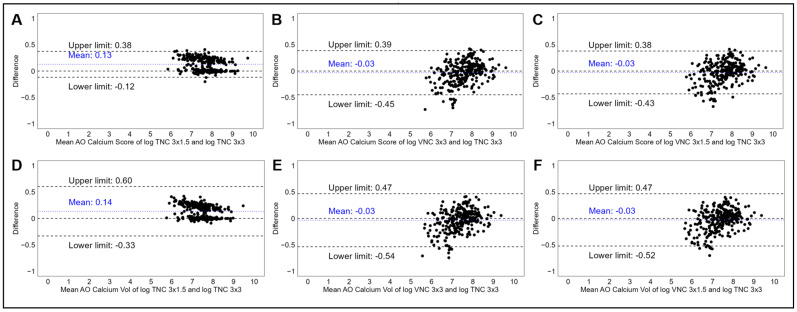
Panel of Bland–Altman plots demonstrating the mean log-transformed AVCS and difference in log-transformed AVCS measurements between TNC 3 × 3 mm and TNC 3 × 1.5 mm (**A**), TNC 3 × 3 mm and VNC 3 × 3 mm (**B**), and TNC 3 × 3 mm and VNC 3 × 1.5 mm (**C**). Bottom panel demonstrating mean log-transformed AVCV and difference in log-transformed AVCV measurements between TNC 3 × 3 mm and TNC 3 × 1.5 mm (**D**), TNC 3 × 3 mm and VNC 3 × 3 mm (**E**), and TNC 3 × 3 mm and VNC 3 × 1.5 mm (**F**). Abbreviations: AVCS, aortic valve calcium score; TNC, true non-contrast; VNC, virtual non-contrast.

Bland–Altman analyses comparing the different acquisition modalities revealed agreement across both TNC and VNC at different slice intervals (Figure 3), with an increase in between-method variability associated with increasing AVC (Supplemental Figures S1 and S2). Compared to AVCS for TNC 3.0 × 3.0 mm, TNC 3.0 × 1.5 mm demonstrated a significantly higher mean AVCS (mean bias 290 ± 418 AU, log-transformed mean bias 0.13 ± 0.13 log AU + 1), reflecting over-estimation. However, Bland–Altman plots reflected very narrow 95% limits of agreement (Figure 3A), with similar results reflected in the analyses of AVCV. Reductions in mean bias were reflected in comparisons with the VNC 3.0 × 3.0 mm and VNC 3.0 × 1.5 mm reconstructions, but increased variability was reflected in wider 95% limits of agreement using log-transformed data (Table 2 and Figure 3B,C).

**Figure 4:**
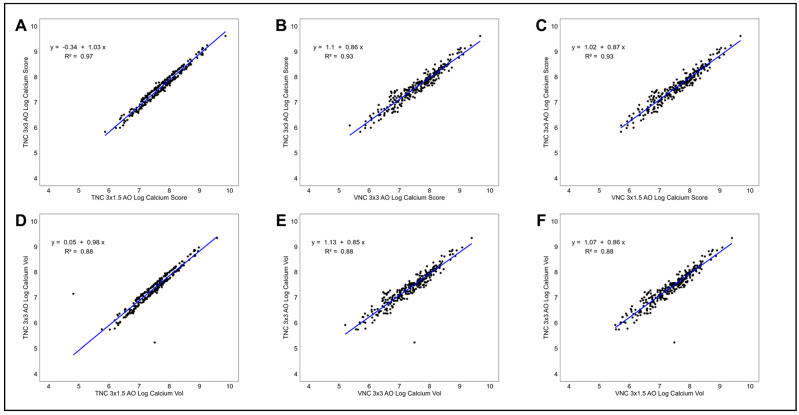
Scatter plots demonstrating comparison in reconstruction methods. Measurement of log-transformed AVCS (**A**–**C**) and AVCV (**D**–**F**) using TNC 3 × 1.5 mm, VNC 3 × 3 mm, and VNC 3 × 1.5 mm, respectively, against the TNC 3 × 3 mm reference standard. Abbreviations: AVC, aortic valve calcium; TNC, true non-contrast; VNC, virtual non-contrast.

Inter-observer agreement for AVCS was excellent across all comparisons, with ICC values ranging from 0.969 (95% CI 0.962–0.975) to 0.971 (95% CI 0.964–0.977). Results were similar when comparing AVCS using log-transformed data and when comparing AVCV across all reconstruction methods. Correction factors generated from linear regressions were estimated to predict TNC 3.0 × 3.0 mm AVCS and AVCV from the alternative reconstruction methods and are displayed in scatter plots (Figure 4), which demonstrated strong linear relationships with excellent model fit.

AVCS was considered to represent severe aortic stenosis when >2000 AU for males and >1200 AU for females. Cohen’s Kappa (κ) was calculated to assess agreement between high/low categories across all reconstructions. Despite the significant difference in mean AVCS comparing TNC 3.0 × 3.0 mm to TNC 1.5 mm, concordance was excellent when comparing high/low categories from these two modalities (κ = 0.81; 95% CI 0.74–0.88), with only 24 (8.6%) subjects misclassified. Similarly, both VNC 3.0 × 3.0 mm and VNC 3.0 × 1.5 mm demonstrated excellent concordance, when high/low categories were compared to that of TNC 3.0 × 3.0 mm (Table 3). The sensitivity and specificity analyses revealed that using TNC 3.0 × 3.0 mm as the reference for AVCS categorization, TNC 3.0 × 1.5 mm had the highest sensitivity (98%; 95% CI 97–100) but the lowest specificity (79%; 95% CI 72–87) out of the three alternate modalities. TNC 3.0 × 1.5 mm also had the greatest negative predictive value (98%; 95% CI 95–100). Both VNC modalities had good sensitivity and greater specificity (Table 4).

### 3.3. Post Hoc Analyses

Post hoc inter- and intra-operator reproducibility analyses demonstrated near perfect reproduction of AVCS and AVCV measurements performed on all four reconstruction modalities and slice thicknesses, with ICC estimates ranging from 0.993 (95% CI 0.978–0.998) to 0.999 (95% CI 0.999–1.00) (Supplemental Table S1). There were no gender differences found when examining stratified Bland–Altman plots (Supplemental Figure S3); stratified ICC estimates were very similar. Although systematic bias was found to be minimal, evidence suggesting proportionality bias reflected in log-transformed Bland–Altman plots were further examined. Statistically significant associations were found after regressing between-measurement differences against mean log-transformed AVC values for each comparison against the TNC 3.0 mm standard, which further suggest a proportional relationship between the subjects’ AVC and differences between reconstruction methods: β = −0.045 SE 0.011 *p* < 0.001; β = 0.116 SE 0.017 *p* < 0.001; and β = 0.104 SE 0.017 *p* < 0.001 for comparisons with TNC 3.0 × 1.5 mm, VNC 3.0 × 3.0 mm, and VNC 3.0 × 1.5 mm, respectively.

## 4. Discussion

This is the largest study to date to directly compare the reliability and accuracy of TNC and VNC imaging for AVCS measurement at different slice thicknesses using PCD-CT. Our findings demonstrate that (1) VNC imaging offers a possible alternative to TNC imaging for AVC quantification, exhibiting excellent agreement across various slice thicknesses; (2) 1.5 mm slice intervals for TNC demonstrated higher AVCS compared to both 3.0 mm TNC and VNC methods at 3.0 mm and 1.5 mm slice intervals; and (3) although TNC 1.5 mm demonstrated the highest sensitivity and NPV, VNC offered both excellent specificities and sensitivities predicting elevated AVCS. The excellent reliability of both modalities, indicated by near-perfect Cohen’s kappa values, supports the potential of VNC imaging to improve clinical workflows and reduce radiation exposure without compromising diagnostic accuracy.

In recent years, the multi-modality approach has highlighted the importance of cardiac CT as a valuable tool for diagnosing aortic stenosis, especially in cases where echocardiography results are inconclusive, with guidelines recommending it as a class IIa indication [15,16]. PCD-CT is a recently approved advanced CT technology that offers improved spatial resolution and allows the creation of VNC images from the available datasets [17]. Several studies have reported excellent reproducibility of VNC images in diagnosis for CACS [18]. However, the consensus on the VNC-generated AVCS in routine clinical application remains to be answered.

The findings of the present study align with the previous literature examining the accuracy of VNC in AVC scoring [19]. Like the Feldle et al. investigation, our study also revealed excellent concordance when comparing the mean AVCS amongst VNC and TNC 3.0 mm slice increment, regardless of slice thickness. However, incorporating the different slice intervals provides valuable insight not addressed in the aforementioned study. Our results highlight a difference in mean AVCS between TNC acquisition at 3.0 mm and 1.5 mm slice intervals, with the TNC 1.5 mm demonstrating notably higher AVCS. This observation is likely attributable to the overlap of segments when 1.5 mm slice interval is used. This is in line with the findings of Boulif et al., which demonstrated the proportional decrease in the calcium AVCS with the increasing slice increment [20].

The high kappa values across all reconstruction methods in the current analysis highlight the strong agreement in AVCS and AVCV classification in severe or non-severe. In contrast, previous studies have suggested significant underestimation when utilizing VNC reconstruction methods [21]. Their study used conventional and pure calcium algorithms that revealed underestimation of AVCS for all the VNC modalities regardless of the algorithm. This difference was more pronounced when conventional algorithm was used compared to pure calcium. In the current analysis, we only synthesized VNC images using the pure calcium algorithm, which produced an insignificant number of cases that underestimated the AVCS compared to the reference (TNC 3.0 mm). Difference in AVCS demonstrated previously may have been due to their higher noise reduction settings (QIR level 3/4), which likely contributed to AVCS underestimation [22]. Our use of lower QIR (level 2) likely helped preserve calcium quantification between TNC and VNC. Other factors contributing to this difference may have been residual contrast interference and VNC reconstructions can also introduce noise or artifacts during the subtraction process, causing variations in calcium assessment. Obtaining images at different keV could also contribute to subtle differences in image reconstruction [23]. Nonetheless, our results show that VNC images are comparable to the standard TNC 3.0 mm.

Our results reveal a trade-off between sensitivity and specificity among the different CT protocols. The TNC 1.5 mm protocol demonstrated the highest sensitivity and NPV but had low specificity, indicating that this modality is most effective for ruling out high AVC. This observation is possible, as thinner slices allow better visualization, whereas minor calcium can be missed on standard 3.0 mm slices, leading to the increased sensitivity that we observed [20]. Our results demonstrated the possibility of proportionality bias owing to the larger between-reconstruction differences with higher AVC scores, suggesting the need to evaluate the precision of AVC measurements at higher values. Meanwhile, the VNC reconstruction exhibited strong balance between sensitivity and specificity, making it more suitable for accurate AVC quantification. Our results presented higher sensitivity and specificity values than previous studies [19]. Such variations can arise from the different patient populations or CT acquisition parameters that can influence the differences. These findings have important clinical ramifications. TNC 1.5 mm may be preferable for screening, while VNC, with higher specificity, might be suitable for precise calcium assessment. There is a need for careful selection of reconstruction techniques based on the clinical context, including a comparison with echocardiographic results and extent of any hypertrophy, which may provide a better sense around the clinical implications of these results.

Our study has several limitations. First, being a single center with a homogenous patient population limits the generalizability of the results. Future studies with larger, multi-center cohorts must validate these results in more diverse patient groups. Second, while our study demonstrated excellent inter- and intra-observer reliability, the AVCS assessment remains subjective to a degree, contributing to differences in image interpretation and score assessment. Third, although the scans were obtained during the motion-free mid-diastolic phase, the HR variability in atrial fibrillation could still theoretically influence the results. This potential impact was not evaluated in our study. Fourth, models which yielded correction factors were not validated for clinical use, and our study did not investigate the prognostic value of these findings. Future studies should include a validation of our correction factors and focus on clinical or procedural outcomes amongst different calcium scores obtained using different modalities.

## 5. Conclusions

Our large, single-center retrospective study confirms the high reproducibility of VNC reconstruction for precise AVC quantification compared to TNC across different slice increments. With an expanded sample size, this study provides more substantial evidence supporting the use of VNC images in clinical practice, potentially streamlining workflows and reducing radiation exposure for patients.

## Figures and Tables

**Table 1:** Patient demographics and CT parameters.

	PCD-CT *
	N = 279
*Demographics*	
Age, year	80 (74, 86)
Female	128 (46)
BMI, kg/m^2^	27.8 (24.5, 31.5)
Hypertension	261 (94)
Hyperlipidemia	213 (76)
Diabetes	92 (33)
History of atrial fibrillation	78 (28)
*CT acquisition parameters*	
Total contrast, mL	70 (60, 70)
Contrast flow rate, mL/s	4 (4, 4)
Peak voltage, kVP	140 ^†^
Total radiation, mSv	18.8 (14.5, 23.7)
*AVC scores*	
Median AVC score, AU	1957 (1181, 2954)
Abnormal ^§^ AVC, n (%)	173 (62)
AVC among male subjects (n = 151)	
Median AVC score, AU	2415 (1742, 3686)
Abnormal ^§^ AVC, n (%)	100 (66)
AVC among female subjects (n = 128)	
Median AVC score, AU	1323 (752, 2239)
Abnormal ^§^ AVC, n (%)	73 (57)

* Data are presented as mean ± standard deviation, median (interquartile range), or number (%). ^†^ Peak voltage set to 140 kVP in all subjects for the contrast study and 120 kVP for the non-contrast images. ^§^ Abnormal AVC defined as >2000 in males or >1200 AU in females. Abbreviations: AVC, aortic valve calcium; BMI, body mass index; CT, computed tomography; PCD-CT, photon-counting detector computed tomography.

**Table 2:** Comparative analysis of aortic valve calcium score and calcium volume in various reconstructions and slice thicknesses using TNC 3.0 mm as a standard reference.

	TNC 3.0 mm, Mean/Median	Comparison	Comparison	Mean Diff	*p*-Value	ICC (95% CI)
		Reconstruction, Median (IQR)	Reconstruction, Mean ± SD			
*Calcium score*						
TNC 3.0 vs. TNC 1.5	2413 ± 1917, 1957 (1181, 2954)	2271 (1347, 3408)	2703 ± 2123	290 ± 418	<0.001	0.969 (0.962, 0.975)
TNC 3.0 vs. VNC 3.0		1954 (956, 3196)	2428 ± 2055	16 ± 485	0.59	0.970 (0.963, 0.976)
TNC 3.0 vs. VNC 1.5		1993 (983, 3180)	2429 ± 2039	16 ± 476	0.57	0.971 (0.964, 0.977)
*Calcium volume*						
TNC 3.0 vs. TNC 1.5	1868 ± 1463, 1536 (951, 2312)	1784 (1058, 2625)	2103 ± 1615	235 ± 336	<0.001	0.965 (0.957, 0.972)
TNC 3.0 vs. VNC 3.0		1524 (764, 2475)	1865 ± 1555	−3 ± 386	0.91	0.967 (0.959, 0.974)
TNC 3.0 vs. VNC 1.5		1545 (807, 2460)	1869 ± 1545	1 ± 377	0.96	0.969 (0.961, 0.975)
*Log-transformed calcium score*						
TNC 3.0 vs. TNC 1.5	7.61 ± 0.68, 7.67 (7.19, 8.04)	7.76 (7.3, 8.15)	7.74 ± 0.65	0.13 ± 0.13	<0.0001	0.964 (0.955, 0.971)
TNC 3.0 vs. VNC 3.0		7.66 (7.01, 8.12)	7.58 ± 0.76	−0.03 ± 0.21	0.0166	0.955 (0.944, 0.964)
TNC 3.0 vs. VNC 1.5		7.67 (7.01, 8.1)	7.59 ± 0.75	−0.03 ± 0.21	0.0397	0.957 (0.946, 0.966)
*Log-transformed calcium volume*						
TNC 3.0 vs. TNC 1.5	7.36 ± 0.68, 7.40 (6.95, 7.77)	7.52 (7.07, 7.89)	7.5 ± 0.65	0.14 ± 0.24	<0.0001	0.916 (0.894, 0.933)
TNC 3.0 vs. VNC 3.0		7.4 (6.77, 7.85)	7.33 ± 0.75	−0.03 ± 0.26	0.0319	0.934 (0.917, 0.947)
TNC 3.0 vs. VNC 1.5		7.41 (6.76, 7.84)	7.33 ± 0.74	−0.03 ± 0.25	0.0785	0.935 (0.919, 0.948)

Abbreviations: TNC, true non-contrast; VNC, virtual non-contrast; ICC, intra-class correlation coefficient; IQR, inter-quartile range; SD, standard deviation.

**Table 3:** Agreement in classification of aortic valve calcium score from different reconstructions techniques and slice thickness using TNC 3.0 as a standard reference.

Aortic Valve Calcium Score * Classification Matrix				Cohens Kappa	Subjects Misclassified
	*TNC 1.5 mm*				
*TNC 3.0 mm*	Low	High	Total	0.81 (0.74–0.88)	24 (8.6%)
Low	84	22	106		
High	2	171	173		
Total	86	193	279		
	*VNC 3.0 mm*				
*TNC 3.0 mm*	Low	High	Total	0.83 (0.76–0.90)	23 (8.2%)
Low	101	5	106		
High	18	155	173		
Total	119	160	279		
	*VNC 1.5 mm*				
*TNC 3.0 mm*	Low	High	Total	0.83 (0.76–0.90)	23 (8.2%)
Low	100	6	106		
High	17	156	173		
Total	117	162	279		

* Presented with point estimate (%) and 95% confidence limits. High AVC score defined as >2000 AU for male and >1200 AU for female subjects. Abbreviations: TNC, true non-contrast; VNC, virtual non-contrast.

**Table 4:** Sensitivity and specificity assessment between different CT reconstruction techniques.

Comparison *	Sensitivity	Specificity	Positive Predictive Value	Negative Predictive Value
TNC 1.5 mm vs. TNC 3.0 mm	98% (97–100)	79% (72–87)	89% (84–93)	98% (95–100)
VNC 3.0 mm vs. TNC 3.0 mm	90% (85–94)	95% (91–99)	97% (94–100)	85% (78–91)
VNC 1.5 mm vs. TNC 3.0 mm	90% (86–95)	94% (90–99)	96% (93–99)	85% (79–92)

* Presented with point estimate (%) and 95% confidence limits. Abbreviations: TNC, true non-contrast; VNC, virtual non-contrast.

## Data Availability

The data presented in this study are available on request from the corresponding author. The data are not publicly available due to privacy restrictions.

## Supplementary Materials

The following supporting information can be downloaded at https://www.mdpi.com/article/10.3390/tomography11120139/s1. Table S1: Inter-observer and Intra-observer coefficient comparing AVC scores and volume between different reconstruction modalities and CT slice thickness; Figure S1: Bland-Altman plots comparing true and virtual non-contrast reconstructions without log transformation; Figure S2: Scatter plots comparing true and virtual non-contrast reconstructions without log transformation; Figure S3: Gender-stratified Bland-Altman plots comparing true and virtual non-contrast reconstructions.

## References

[1] Scalia I.G., Farina J.M., Padang R., Jokerst C.E., Pereyra M., Mahmoud A.K., Naqvi T.Z., Chao C.-J., Oh J.K., Arsanjani R. (2023). Aortic Valve Calcium Score by Computed Tomography as an Adjunct to Echocardiographic Assessment—A Review of Clinical Utility and Applications. J. Imaging.

[2] Tastet L., Ali M., Pibarot P., Capoulade R., Øvrehus K.A., Arsenault M., Haujir A., Bédard É., Diederichsen A.C.P., Dahl J.S. (2024). Grading of Aortic Valve Calcification Severity and Risk Stratification in Aortic Stenosis. J. Am. Heart Assoc..

[3] Otto C.M., Nishimura R.A., Bonow R.O., Carabello B.A., Erwin J.P., Gentile F., Jneid H., Krieger E.V., Mack M., McLeod C. (2021). 2020 ACC/AHA Guideline for the Management of Patients With Valvular Heart Disease: A Report of the American College of Cardiology/American Heart Association Joint Committee on Clinical Practice Guidelines. Circulation.

[4] Vahanian A., Beyersdorf F., Praz F., Milojevic M., Baldus S., Bauersachs J., Capodanno D., Conradi L., De Bonis M., De Paulis R. (2022). 2021 ESC/EACTS Guidelines for the management of valvular heart disease. Eur. Heart J..

[5] Flohr T., Schmidt B., Ulzheimer S., Alkadhi H. (2023). Cardiac imaging with photon counting CT. Br. J. Radiol..

[6] Pawade T., Sheth T., Guzzetti E., Dweck M.R., Clavel M.-A. (2019). Why and How to Measure Aortic Valve Calcification in Patients With Aortic Stenosis. JACC: Cardiovasc. Imaging.

[7] Vecsey-Nagy M., Varga-Szemes A., Emrich T., Zsarnoczay E., Nagy N., Fink N., Schmidt B., Nowak T., Kiss M., Vattay B. (2023). Calcium scoring on coronary computed angiography tomography with photon-counting detector technology: Predictors of performance. J. Cardiovasc. Comput. Tomogr..

[8] von Elm E., Altman D.G., Egger M., Pocock S.J., Gotzsche P.C., Vandenbroucke J.P., Initiative S. (2008). The Strengthening the Reporting of Observational Studies in Epidemiology (STROBE) statement: Guidelines for reporting observational studies. J. Clin. Epidemiol..

[9] Guzzetti E., Oh J.K., Shen M., Dweck M.R., Poh K.K., Abbas A.E., Mando R., Pressman G.S., Brito D., Tastet L. (2022). Validation of aortic valve calcium quantification thresholds measured by computed tomography in Asian patients with calcific aortic stenosis. Eur. Heart J.-Cardiovasc. Imaging.

[10] Raunig D.L., McShane L.M., Pennello G., Gatsonis C., Carson P.L., Voyvodic J.T., Wahl R.L., Kurland B.F., Schwarz A.J., Gönen M. (2015). Quantitative imaging biomarkers: A review of statistical methods for technical performance assessment. Stat. Methods Med. Res..

[11] Bland J.M., Altman D.G. (1986). Statistical methods for assessing agreement between two methods of clinical measurement. Lancet.

[12] Clavel M.A., Messika-Zeitoun D., Pibarot P., Aggarwal S.R., Malouf J., Araoz P.A., Michelena H.I., Cueff C., Larose E., Capoulade R. (2013). The complex nature of discordant severe calcified aortic valve disease grading: New insights from combined Doppler echocardiographic and computed tomographic study. J. Am. Coll. Cardiol..

[13] Clavel M.A., Pibarot P., Messika-Zeitoun D., Capoulade R., Malouf J., Aggarval S., Araoz P.A., Michelena H.I., Cueff C., Larose E. (2014). Impact of aortic valve calcification, as measured by MDCT, on survival in patients with aortic stenosis: Results of an international registry study. J. Am. Coll. Cardiol..

[14] Bland J.M., Altman D.G. (1999). Measuring agreement in method comparison studies. Stat. Methods Med. Res..

[15] Isselbacher E.M., Preventza O., Hamilton Black J., Augoustides J.G., Beck A.W., Bolen M.A., Braverman A.C., Bray B.E., Brown-Zimmerman M.M., Chen E.P. (2022). 2022 ACC/AHA Guideline for the Diagnosis and Management of Aortic Disease: A Report of the American Heart Association/American College of Cardiology Joint Committee on Clinical Practice Guidelines. Circulation.

[16] Dweck M.R., Loganath K., Bing R., Treibel T.A., McCann G.P., Newby D.E., Leipsic J., Fraccaro C., Paolisso P., Cosyns B. (2023). Multi-modality imaging in aortic stenosis: An EACVI clinical consensus document. Eur. Heart J.-Cardiovasc. Imaging.

[17] Dirrichs T., Schröder J., Frick M., Huppertz M., Iwa R., Allmendinger T., Mecking I., Kuhl C.K. (2024). Photon-Counting Versus Dual-Source CT for Transcatheter Aortic Valve Implantation Planning. Acad. Radiol..

[18] Emrich T., Aquino G., Schoepf U.J., Braun F.M., Risch F., Bette S.J., Woznicki P., Decker J.A., O’Doherty J., Brandt V. (2022). Coronary Computed Tomography Angiography-Based Calcium Scoring: In Vitro and In Vivo Validation of a Novel Virtual Noniodine Reconstruction Algorithm on a Clinical, First-Generation Dual-Source Photon Counting-Detector System. Investig. Radiol..

[19] Feldle P., Scheuber M., Grunz J.-P., Heidenreich J.F., Pannenbecker P., Nora C., Huflage H., Bley T.A., Petritsch B. (2024). Virtual non-iodine photon-counting CT-angiography for aortic valve calcification scoring. Sci. Rep..

[20] Boulif J., Gerber B., Slimani A., Lazam S., de Meester C., Piérard S., Pasquet A., Pouleur A.C., Vancraeynest D., El Khoury G. (2017). Assessment of aortic valve calcium load by multidetector computed tomography. Anatomical validation, impact of scanner settings and incremental diagnostic value. J. Cardiovasc. Comput. Tomogr..

[21] Risch F., Harmel E., Rippel K., Wein B., Raake P., Girdauskas E., Elvinger S., Owais T., Scheurig-Muenkler C., Kroencke T. (2024). Virtual non-contrast series of photon-counting detector computed tomography angiography for aortic valve calcium scoring. Int. J.-Cardiovasc. Imaging.

[22] Fink N., Emrich T., Schoepf U.J., Zsarnoczay E., O’Doherty J., Halfmann M.C., Griffith J.P., Pinos D., Suranyi P., Baruah D. (2024). Improved Detection of Small and Low-Density Plaques in Virtual Noncontrast Imaging-based Calcium Scoring at Photon-Counting Detector CT. Radiol. Cardiothorac. Imaging.

[23] Fink N., Zsarnoczay E., Schoepf U.J., Griffith J.P., Wolf E.V., O’Doherty J., Suranyi P., Baruah D., Kabakus I.M., Ricke J. (2023). Photon Counting Detector CT-Based Virtual Noniodine Reconstruction Algorithm for In Vitro and In Vivo Coronary Artery Calcium Scoring: Impact of Virtual Monoenergetic and Quantum Iterative Reconstructions. Investig. Radiol..

