# Supplementary material for: Aortic Valve Calcium Scoring Using True and Virtual Non-Contrast Reconstructions on Photon-Counting CT with Differing Slice Increments: Impact on Calcium Severity Classifications
Source: Tomography. 2025 Dec 11;11(12):139. doi: 10.3390/tomography11120139 (PMC12736715; doi:10.3390/tomography11120139)
Supplement: Supplementary file 1 [file tomography-11-00139-s001.zip › tomography-3962026-supplementary.pdf]

Supplementary Appendix

Supplement to: Singh M, et al., Aortic Valve Calcium Scoring Using True and Virtual Non-Contrast Reconstructions on Photon Counting CT with Differing Slice Increment: Impact on Calcium Severity Classifications

Table of Contents

		Page
Supplemental Table S1.	Inter-observer and Intra-observer coefficient	2
Supplemental Figure S1.	Bland-Altman plots comparing true and virtual non-contrast reconstructions without log transformation	3
Supplemental Figure S2.	Scatter plots comparing true and virtual non-contrast reconstructions without log transformation	4
Supplemental Figure S3.	Gender-stratified Bland-Altman plots comparing true and virtual non-contrast reconstructions	5

Supplemental Table S1: Inter-observer and Intra-observer coefficient comparing AVC scores and volume between different reconstruction modalities and CT slice thickness

**Inter observer assessment**					
Measurement Type	Mean of User 1	Mean of User 2	Mean Difference	p-value	ICC
TNC 1.5 mm AVC score	2842.85 ± 2814.23	2850.24 ± 2847.06	-7.386 ± 54.97	0.62	0.9998 (0.9995, 0.9999)
TNC 1.5 mm AVC volume	2202.4 ± 2137.98	2204.69 ± 2168.68	-2.286 ± 45.78	0.85	0.9998 (0.9995, 0.9999)
TNC 3.0 mm AVC score	2906.29 ± 2899.9	2867.16 ± 2817.33	39.129 ± 136.83	0.3	0.9988 (0.9967, 0.9996)
TNC 3.0 mm AVC volume	2244.08 ± 2192.54	2207.82 ± 2131.16	36.257 ± 111.43	0.25	0.9985 (0.9957, 0.9995)
VNC 1.5 mm AVC score	2734.59 ± 2905.43	2738.97 ± 2916.41	-4.386 ± 13.18	0.24	0.9999 (0.9996, 1.00)
VNC 1.5 mm AVC volume	2095.45 ± 2202.19	2102.56 ± 2220.29	-7.107 ± 20.4	0.21	0.9999 (0.9996, 1.00)
VNC 3.0 mm AVC score	2734.73 ± 2892.94	2745.8 ± 2919.35	-11.071 ± 39.32	0.31	0.9999 (0.9996, 1.00)
VNC 3.0 mm AVC volume	2100.27 ± 2196.08	2111.19 ± 2224.54	-10.914 ± 33.01	0.23	0.9999 (0.9998, 0.9999)
**Intra observer assessment**					
Measurement Type	Mean of Measurement 1	Mean of Measurement 2	Mean Difference	p-value	ICC
TNC 1.5 mm AVC score	3485.72 ± 2266.52	3548.04 ± 2232.42	-62.32 ± 253.48	0.36	0.9933 (0.9782, 0.9979)
TNC 1.5 mm AVC volume	2749.63 ± 1691.14	2749.29 ± 1690.38	0.34 ± 38.69	0.97	0.9997 (0.999, 0.9999)
TNC 3.0 mm AVC score	3300.73 ± 2206.45	3298.56 ± 2199.19	2.167 ± 112.84	0.94	0.9987 (0.9957, 0.9996)
TNC 3.0 mm AVC volume	2556.78 ± 1669.6	2553.03 ± 1659.76	3.753 ± 92.42	0.88	0.9985 (0.995, 0.9995)
VNC 1.5 mm AVC score	3129.23 ± 2164.26	3114.99 ± 2121.61	14.247 ± 89.12	0.55	0.9991 (0.9974, 0.9997)
VNC 1.5 mm AVC volume	2443.25 ± 1632.84	2433.03 ± 1601.86	10.22 ± 70.77	0.58	0.999 (0.997, 0.9997)
VNC 3.0 mm AVC score	3181.04 ± 2150.99	3163.29 ± 2147.23	17.747 ± 118.53	0.57	0.9984 (0.9948, 0.9995)
VNC 3.0 mm AVC volume	2428.38 ± 1618.02	2445.28 ± 1620.52	-16.9 ± 105.46	0.54	0.9978 (0.9928, 0.9993)

Abbreviations: TNC, true non-contrast; VNC, virtual non-contrast; ICC, inter- or intra- class correlation coefficient

Supplemental Figure S1. Bland-Altman plots comparing true and virtual non-contrast reconstructions without log transformation

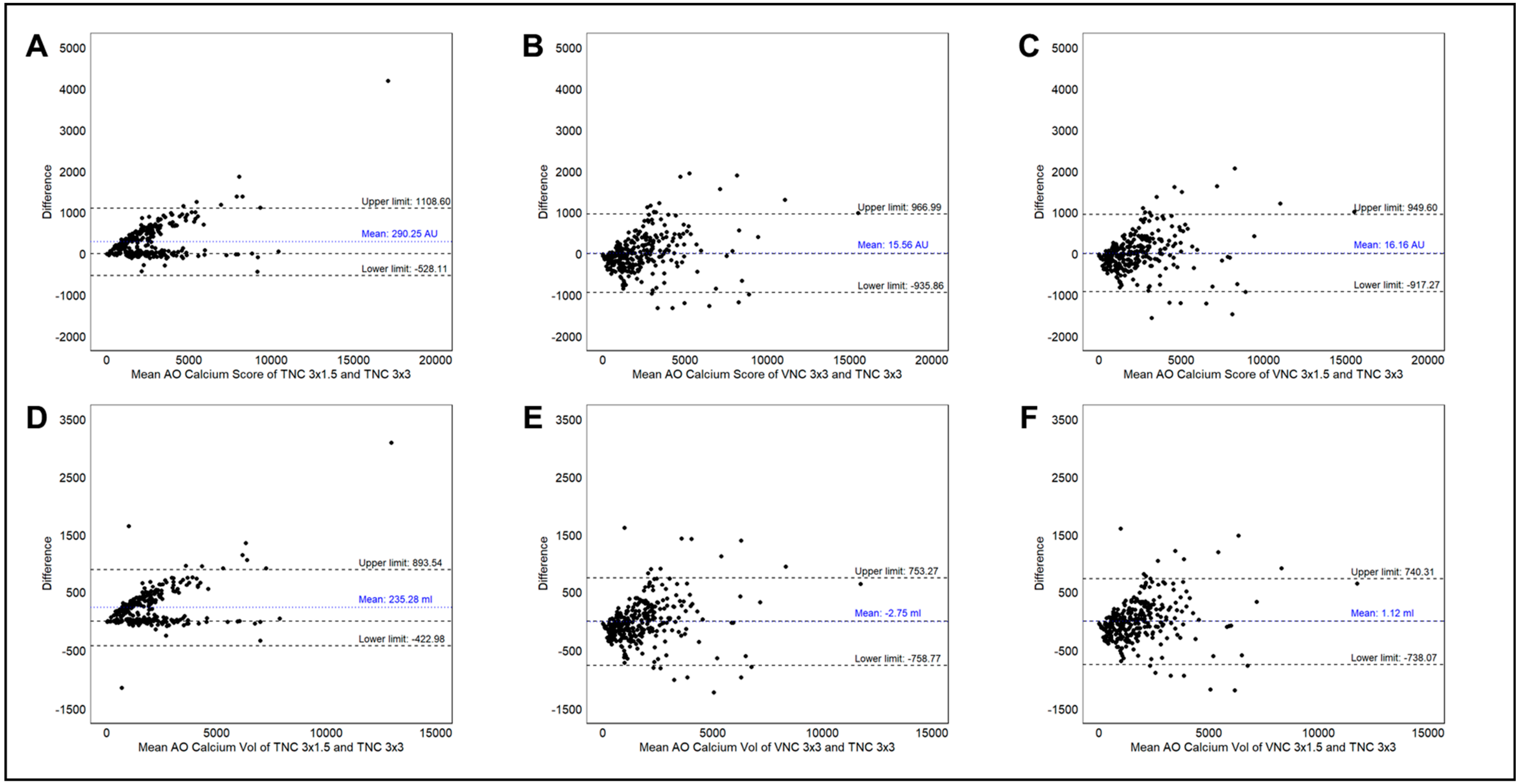

Panel of Bland-Altman plots demonstrating the mean AVCS and difference in AVCS measurement between TNC 3x3 mm and TNC 3x1.5mm (A), TNC 3x3 mm and VNC 3x3 mm (B), and TNC 3x3 mm and VNC 3x1.5 mm (C). Bottom Panel demonstrating mean AVC volume and difference in AVC volume measurement between TNC 3x3 mm and TNC 3x1.5mm (D), TNC 3x3 mm and VNC 3x3 mm (E), and TNC 3x3 mm and VNC 3x1.5 mm (F).

Supplemental Figure S2. Scatter plots comparing true and virtual non-contrast reconstructions without log transformation

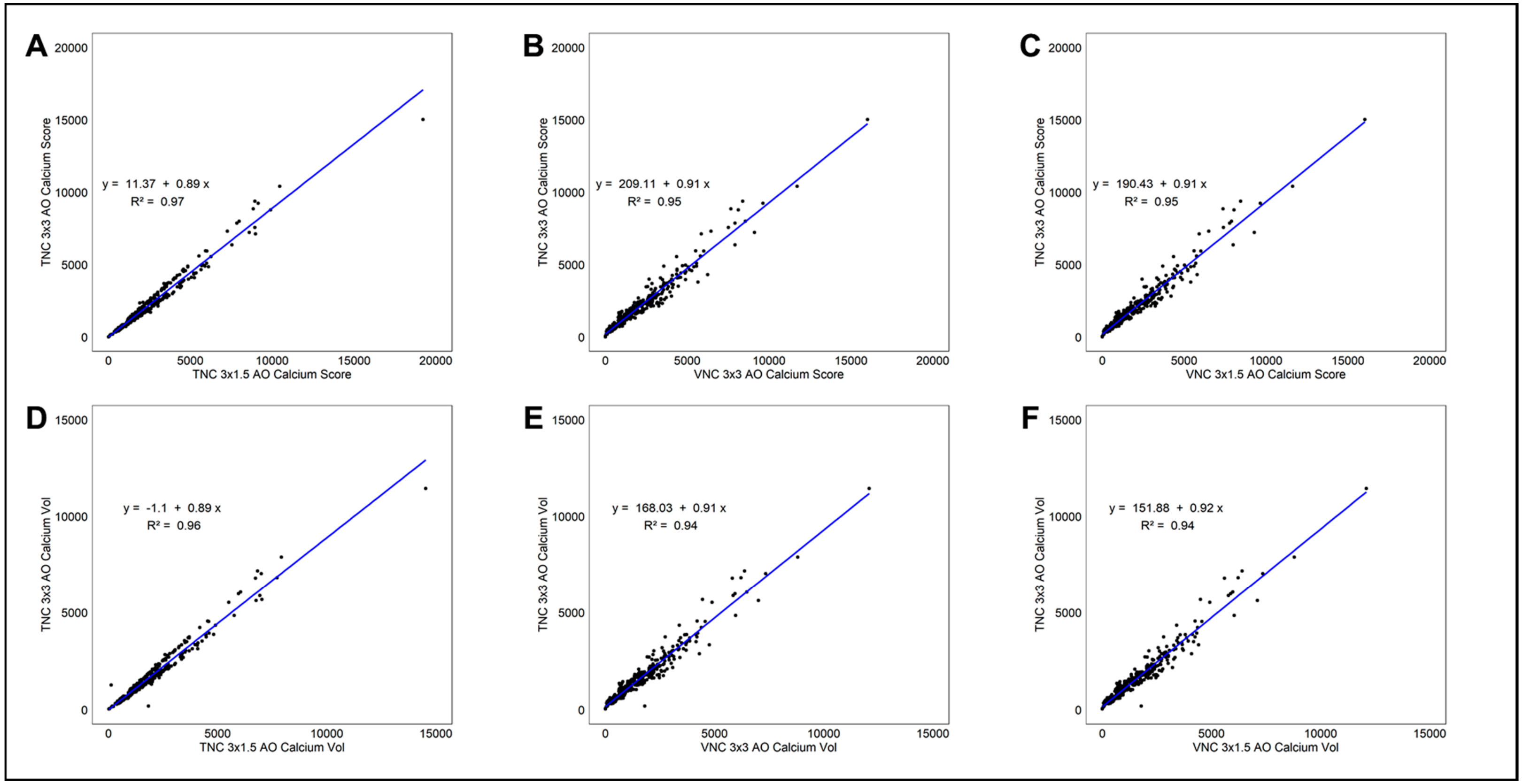

Scatter plots demonstrating comparison in reconstruction methods measuring AVC score (A, B, C) and AVC volume (D, E, F) using TNC 3x1.5mm, VNC 3x3 mm, and VNC 3x1.5 mm, respectively, against the TNC 3x3 mm reference standard.

Supplemental Figure S3. Gender-stratified Bland-Altman plots comparing true and virtual non-contrast reconstructions

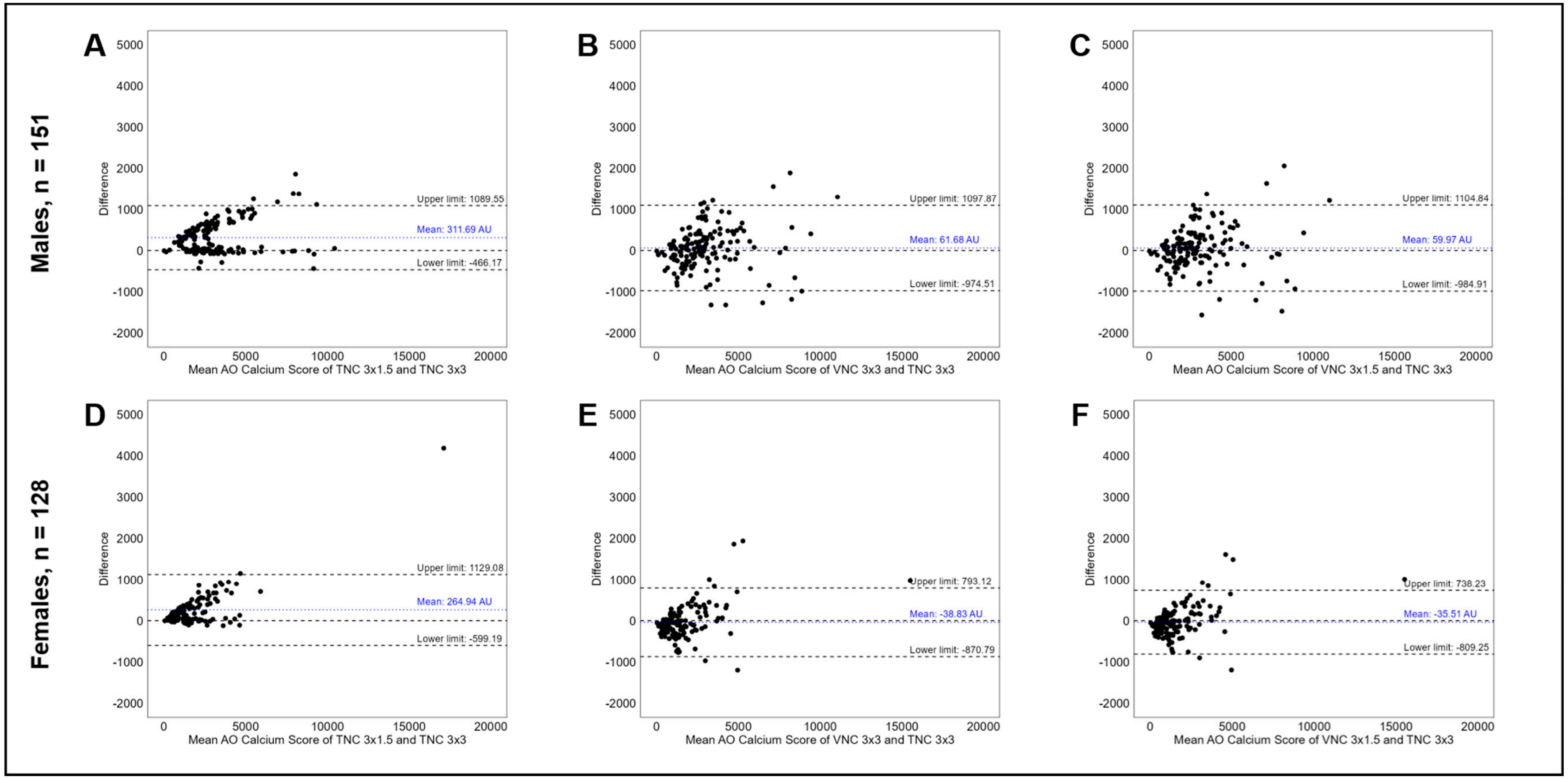

Legend: Panel of Bland-Altman plots demonstrating the AVCS variability among males, n = 151 (top row) and females, n = 128 (bottom row) between the following reconstructions: TNC 3x3 mm and TNC 3x1.5mm (A, D), TNC 3x3 mm and VNC 3x3 mm (B, E), and TNC 3x3 mm and VNC 3x1.5 mm (C, F).
